# Human organoids are superior to cell culture models for intestinal barrier research

**DOI:** 10.3389/fcell.2023.1223032

**Published:** 2023-10-02

**Authors:** Catherine Kollmann, Hannah Buerkert, Michael Meir, Konstantin Richter, Kai Kretzschmar, Sven Flemming, Matthias Kelm, Christoph-Thomas Germer, Christoph Otto, Natalie Burkard, Nicolas Schlegel

**Affiliations:** ^1^ Department of General, Visceral, Transplant, Vascular and Pediatric Surgery, University Hospital Wuerzburg, Wuerzburg, Germany; ^2^ Mildred-Scheel Early Career Centre (MSNZ) for Cancer Research, University Hospital Wuerzburg, Wuerzburg, Germany

**Keywords:** intestinal epithelial barrier, Caco2 cells, intestinal organoids, enteroids, gut barrier, inflammatory cell model, inflammation

## Abstract

Loss of intestinal epithelial barrier function is a hallmark in digestive tract inflammation. The detailed mechanisms remain unclear due to the lack of suitable cell-based models in barrier research. Here we performed a detailed functional characterization of human intestinal organoid cultures under different conditions with the aim to suggest an optimized *ex-vivo* model to further analyse inflammation-induced intestinal epithelial barrier dysfunction. Differentiated Caco2 cells as a traditional model for intestinal epithelial barrier research displayed mature barrier functions which were reduced after challenge with cytomix (TNFα, IFN-γ, IL-1ß) to mimic inflammatory conditions. Human intestinal organoids grown in culture medium were highly proliferative, displayed high levels of LGR5 with overall low rates of intercellular adhesion and immature barrier function resembling conditions usually found in intestinal crypts. WNT-depletion resulted in the differentiation of intestinal organoids with reduced LGR5 levels and upregulation of markers representing the presence of all cell types present along the crypt-villus axis. This was paralleled by barrier maturation with junctional proteins regularly distributed at the cell borders. Application of cytomix in immature human intestinal organoid cultures resulted in reduced barrier function that was accompanied with cell fragmentation, cell death and overall loss of junctional proteins, demonstrating a high susceptibility of the organoid culture to inflammatory stimuli. In differentiated organoid cultures, cytomix induced a hierarchical sequence of changes beginning with loss of cell adhesion, redistribution of junctional proteins from the cell border, protein degradation which was accompanied by loss of epithelial barrier function. Cell viability was observed to decrease with time but was preserved when initial barrier changes were evident. In summary, differentiated intestinal organoid cultures represent an optimized human *ex-vivo* model which allows a comprehensive reflection to the situation observed in patients with intestinal inflammation. Our data suggest a hierarchical sequence of inflammation-induced intestinal barrier dysfunction starting with loss of intercellular adhesion, followed by redistribution and loss of junctional proteins resulting in reduced barrier function with consecutive epithelial death.

## 1 Introduction

The intestinal epithelial barrier (IEB) protects the inner part of the body against luminal microbiota and antigens while permitting trans- or paracellular transport of water, nutrients and selected molecules (Luissint et al., 2016; Schlegel et al., 2021). The IEB consists of columnar epithelial cells that line the gastrointestinal tract as a single monolayer that undergoes a permanent renewal starting from the crypts where intestinal epithelial stem cells proliferate and then differentiate along the crypt-villus axis into different cell types that are predominantly enterocytes. The intercellular space between epithelial cells is sealed by the apical junctional complex, which consists of tight junctions, adherens junctions and desmosomes (Farquhar and Palade, 1963; Schlegel et al., 2021). Sealing of the intercellular cleft is secured by tight junctions that consist of claudins and Occludin (OCLN), while intercellular adhesion is provided by adherens junctions and desmosomes (Farquhar and Palade, 1963; Luissint et al., 2016; Schlegel et al., 2021). There is increasing evidence that these junctional proteins regulate each other to form a functional unit that controls IEB integrity (Rübsam et al., 2018; Burkard et al., 2021).

Under conditions of intestinal inflammation, paracellular permeability is increased and thereby contributes to the development, perpetuation and severity of digestive tract inflammation. This is typically seen in patients suffering from inflammatory bowel diseases (IBD) (Martini et al., 2017). In addition, loss of cell viability and changes of the differentiation pattern contribute to the loss of barrier functions in acute and chronic inflammation (Zheng et al., 2011; Koch and Nusrat, 2012; Neurath, 2014a; Coskun, 2014). The detailed mechanisms are incompletely understood. Although several cellular events including loss of intercellular adhesion, reduction of junctional proteins and epithelial cell death have been described in intestinal inflammation, it remains unclear whether all of these occur simultaneously and independent or follow a hierarchical sequence (Koch and Nusrat, 2012). This is clinically relevant since mucosal healing and restoration of the IEB are essential for clinical remission and improvement of patients’ outcomes in digestive tract inflammation including IBD, making it a promising target for new therapies (Neurath and Travis, 2012; Neurath, 2014b; Okamoto and Watanabe, 2016; Chang et al., 2017).

Most studies about cellular mechanisms of intestinal barrier regulation have been conducted in cell cultures that derived from colon carcinoma so far. With the implementation of intestinal organoid cultures from human intestinal stem cells (Sato et al., 2009) there is an unrivalled *ex-vivo* model available that allows to investigate numerous epithelial disorders in healthy primary human epithelial cells. Using explicit differentiation protocols, intestinal organoids can be diversified into the different cell types that are also identified in the human intestinal epithelium (Yin et al., 2014). The use of organoids to further investigate the cellular mechanisms of intestinal barrier dysfunction has been proposed earlier (Yoo and Donowitz, 2019; Meir et al., 2020; Schoultz and Keita, 2020). However, despite sophisticated reports of transcriptomic changes of organoids under various conditions, there is no detailed description that allows the reproducible assessment of intestinal epithelial barrier properties in functional assays and therefore still most researchers use immortalized intestinal cancer-derived cell lines for intestinal barrier research. To improve the cell-based model systems in intestinal barrier research we hypothesized that human intestinal organoids are a superior tool to investigate inflammation-induced changes of IEB compared to conventional cell culture models. Therefore, we performed detailed analyses and comparisons using conventional differentiated Caco2 cell cultures and human intestinal organoids to identify changes in IEB integrity following different inflammatory challenges.

## 2 Materials and methods

### 2.1 Caco2 cell culture

Caco2 cells (ATCC, LGC Standards, Wesel, Germany) were cultured in Dulbecco’s Modified Eagle’s Medium (DMEM; Sigma-Aldrich, St. Louis, MO, United States) supplemented with 100 U/mL Penicillin-Streptomycin (Gibco, Thermo Fisher Scientific, Waltham, MA, United States) and 10% fetal calf serum (FCS; Gibco) (DMEM++). For passaging, cells were incubated with 1% ethylenediaminetetraacetic acid (EDTA; Serva Electrophoresis, Heidelberg, Germany) in Dulbecco’s Phosphate Buffered Saline (PBS; Gibco) for 10–12 min at 37°C when reaching at least 80% confluency before dissolving the monolayer through incubation with Trypsin-EDTA solution (Sigma-Aldrich) for 2–3 min at 37°C. Trypsinization was stopped by adding adequate amount of DMEM before cell aggregations were split mechanically by pipetting up and down thoroughly with the Pasteur pipette. After centrifugation at 300 g for 3 min at room temperature, supernatant was discarded and the pellet dissolved in DMEM++ to seed for further culture (1:10 to 1:20) or experiments (Schlegel et al., 2010; Meir et al., 2015; Spindler et al., 2015; Meir et al., 2019; Burkard et al., 2021; Meir et al., 2021; Nagler et al., 2022).

Caco2 cells were cultured and treated in Cellstar multiwell plates (Greiner Bio-One, Kremsmuenster, Austria) for Western blot analyses, on 12 well chambered coverslips (Ibidi, Graefelfing, Germany) for immunofluorescence staining or on electrode arrays (Applied Biophysics, Troy, NY, United States) or ThinCert cell culture inserts (Greiner Bio-One) for transepithelial electrical resistance (TER) measurements. Experiments were conducted when reaching confluence following 24 h of serum-starvation (Schlegel et al., 2010; Meir et al., 2019; Burkard et al., 2021; Nagler et al., 2022).

### 2.2 Human intestinal organoid culture

#### 2.2.1 Ethical approval

Prior to surgery, all patients gave written informed consent for sample collection. This was obtained via the broad consent of the medical faculty of the University of Wuerzburg. The Ethical Board of the University of Wuerzburg gave ethical approval (proposal numbers 46/11, 113/13, 42/16, amendment 2020, 171/22) for this study.

#### 2.2.2 Isolating intestinal stem cells

Human full-wall tissue samples were obtained from patients with an indication for surgical bowel resection (e.g., for colon carcinoma) from a healthy resection margin (small intestine or colon). Tissue samples were immediately placed in Hank’s Balanced Salt Solution (HBSS; Sigma-Aldrich) supplemented with 1% Antibiotic-Antimycotic 100x (Gibco) (HBSS/a-a) for transportation on ice. After washing the sample three times with PBS, the mucosa was dissected, mucus and villi were scraped off gently with glass slides and the mucosa was cut into strips. The mucosa was washed in HBSS/a-a by vortexing 5 s and then digested in HBSS/a-a supplemented with 2 mM EDTA under constant rotation for 30 min at 4°C. After digestion, the mucosa was washed by inverting slowly in fresh HBSS/a-a. The mucosa was then transferred into 10 mL HBSS/a-a supplemented with 1% Gentamicin (Genaxxon bioscience, Ulm, Germany) and gently shaken for 10 s to extract crypts. This process was repeated two more times with new tubes and increasing shaking force and duration. Each supernatant was checked for adequate number of crypts by microscopy and the supernatant with the highest number of crypts was centrifuged at 300 g for 3 min. The pellet was resuspended in 1 mL basal medium (BM) consisting of Advanced DMEM/F-12 (Gibco) supplemented with 1% Antibiotic-Antimycotic 100x, 1% N-2 (100x), 2% B-27 (50x) w/o Vitamin A, 10 mM 4-(2-hydroxyethyl)-1-piperazineethanesulfonic acid (HEPES), 2 mM GlutaMAX (all from Gibco) and 1 mM N-Acetyl-L-cysteine (Sigma-Aldrich) and centrifuged again at 300 g for 3 min at room temperature. The pellet was resuspended in 4°C cold mixture of 50% Growth Factor Reduced Basement Membrane Matrix (Corning, Corning, NY, United States) plus 50% IntestiCult Organoid Growth Medium Human (Stemcell Technologies, Vancouver, Canada) and seeded as 5–10 µL droplets in Cellstar multiwell plates. After the gel mixture solidified upside down for 20–30 min at 37°C, the plate was reversed and 37°C warm IntestiCult supplemented with 10 µM Y-27632 dihydrochloride (Tocris Bioscience, Bio-Techne, Bristol, United Kingdom) was added. Medium was changed every 2–3 days using IntestiCult (Meir et al., 2019; Meir et al., 2021; Nagler et al., 2022). In this project we used organoid cell lines from 8 different patients at different passages.

#### 2.2.3 Passaging of intestinal organoids

By rinsing the well with its own medium, gel droplets were destructed and the intestinal organoids harvested. In case of larger gel remains on the plate, it was rinsed again with PBS. To extract the intestinal organoids, the solution was centrifuged at 300 g for 3 min at room temperature and the supernatant discarded. Washing of the intestinal organoids was executed by dissolving the pellet in 1mL BM, centrifugation at 300 g for 3 min and discarding the supernatant. For mechanical splitting, the pellet was resuspended in 1 mL TypLE Express Enzyme (Gibco) and pipetted 10–20x strongly up and down with a 10 µL unstuffed pipette tip on top of a 1,000 µL pipette tip (double tip). To stop the splitting process, 1 mL BM was added before the solution was centrifuged at 300 g for 3 min at room temperature and the supernatant discarded. The pellet was washed again as described above and then seeded as described above for further culture or experiments.

#### 2.2.4 2D monolayer culture

Three-dimensional (3D) intestinal organoids were transformed into a two-dimensional (2D) culture for immunofluorescence staining and measurements of TER. The organoids were collected as described above for passaging of the intestinal organoids. After washing with BM, the pellet was resuspended in 1 mL TrypLE Express Enzyme and kept at 37°C for approximately 5 min. Then, the solution was pipetted up and down strongly with the double tip as described above to create a single cell solution. This process was monitored by repetitive microscopy. To stop the splitting process, 1 mL BM was added and the sample washed again with BM as described above before resuspending the cells in IntestiCult/Y-27632. 2D cultures were established on 12 well chambered coverslips, electrode arrays, ThinCert cell culture inserts or Cellstar multiwell plates. To create a coating for 2D culture, the respective equipment was covered in 10% Geltrex LDEV-Free Reduced Growth Factor Basement Membrane Matrix (Gibco) in BM at room temperature. After 30 min the mixture was removed to let the surface dry before seeding the single cell solution in IntestiCult/Y-27632. Culture medium was added to the apical compartment of ThinCert cell culture insert for culture purposes and test reagents were added to both the apical and the basal compartments to mimic physiological conditions.

#### 2.2.5 Differentiation of intestinal organoids

To initiate differentiation in proliferating intestinal organoids, they were subjected to Wingless-related integration site (WNT)-depletion. Differentiation medium consists of BM supplemented with 500 ng/mL hR-Spondin-1, 100 ng/mL mNoggin, 50 ng/mL human epidermal growth factor (all from PeproTech, Hamburg, Germany), 10 mM Nicotinamide, 10µM SB202190, 10 nM [Leu15]-Gastrin I human (all from Sigma-Aldrich), 500 nM A83-01 (Tocris Bioscience), 500 nM LY2157299 (Axon MedChem, Groningen, Netherlands) for the duration of 4 days.

### 2.3 Experiments

#### 2.3.1 Test reagents and cytokines

To generate an inflammatory phenotype, Caco2 cells and intestinal organoids were incubated with cytomix, a composition of 100 ng/mL human Tumor necrosis Factor-α (TNFα; Merck Millipore, Burlington, MA, United States), 10 ng/mL human Interleukin 1β (IL-1β; Sigma-Aldrich) and 50 ng/mL human Interferon-γ (IFNγ; Merck Millipore) in DMEM or differentiation medium respectively (Meir et al., 2015; Meir et al., 2019; Burkard et al., 2021; Meir et al., 2021). The respective culture medium was replaced with cytomix and incubated at 37°C for at least 4 and up to 24 h.

#### 2.3.2 Western blot

After washing with PBS, Caco2 cells were harvested in lysis buffer containing 25 mM HEPES (Gibco), 2 mM EDTA, 25 mM Sodium fluoride (Sigma-Aldrich), 1% sodium dodecyl sulfate (SDS; Carl Roth, Karlsruhe, Germany) and 1% Protease Inhibitor Cocktail 100x (Thermo Scientific, Thermo Fisher Scientific, Waltham, MA, United States) and stored at −20°C. The intestinal organoids were collected as described above. The solution was kept on ice for 15–30 min to allow the gel to dissolve completely. After centrifuging at 300 g for 3 min and discarding the supernatant, the pellet was resuspended in 1 mL 4°C cold sterile PBS. The samples were transferred to a 1.5 mL tube and centrifuged at 500 rpm for 5min before the pellet was resuspended in lysis buffer and stored at −20°C.

After sonicating the samples, protein amount in cell lysates was computed by Pierce BCA Protein Assay Kit (Thermo Scientific) according to the manufacturer’s instructions. Cell lysates were mixed with 4x Laemmli buffer consisting of 25 mL 1 M Tris ultrapure (PanReac AppliChem, Darmstadt, Germany) in distilled water which was pH adjusted to 6.8 by titrating hydrochloric acid (Carl Roth) plus 40 mL Glycerol (Sigma-Aldrich) and 8 g SDS. Proteins were separated using SDS-polyacrylamide gel electrophoresis before being transferred to a nitrocellulose membrane (Thermo Scientific) as described before (Ungewiß et al., 2017; Gross et al., 2018). After blocking with 5% nonfat dried milk powder (PanReac AppliChem) in TBST buffer containing 5 mM Tris ultrapure, 15 mM Sodium chloride (Sigma-Aldrich) and 0.5% Tween 20 (PanReac AppliChem), membranes were incubated with primary antibodies as outlined in Supplementary Table S1. Then, species specific peroxidase-conjugated secondary antibodies were applied and visualized with SuperSignal West Pico PLUS Chemiluminescent Substrate (Thermo Scientific) based imaging with ChemicDoc Touch Imaging System (Bio-Rad Laboratories, Hercules, CA, United States). Chemiluminescence signals were quantified as optical densities (OD) using Image Lab (Bio-Rad Laboratories) for statistical calculation in relation to β-actin (Meir et al., 2015; Burkard et al., 2021; Meir et al., 2021).

#### 2.3.3 Immunostaining

2D Caco2 cells and intestinal organoids were used for immunofluorescence staining while intestinal organoids were also cultured in their 3D form for paraffin embedding. For this, intestinal organoids were harvested and washed as described for Western blot analysis. The pellet was then resuspended in formaldehyde solution 3.5% (PFA; Otto Fischar, Saarbruecken, Germany). After 10 min at room temperature, the sample was centrifuged at 300 g for 3 min, the supernatant discarded and the pellet washed with 1 mL sterile PBS as described above. The pellet was then resuspended in 37°C prewarmed HistoGel (Thermo Scientific) and immediately pipetted onto 4°C cold glass slides on ice. After 1 min the HistoGel solidified which allowed transfer into the embedding cassette. The sample was again fixed in PFA and embedded in paraffin.

Formalin-fixed, paraffin-embedded sections on glass slides were deparaffinized in Xylene AnalaR Normapur (VWR International, Radnor, PA, United States) and hydrated by descending ethanol (Carl Roth) solutions in distilled water and finally in PBS. Proteins were retrieved by boiling the sections in 10 mM Tri-Na-Citrat-Dihydrat (PanReac AppliChem) for 10min. Cultured 2D cell monolayers were grown to confluence on chambered coverslips. After washing with PBS, cells were fixed by incubation with PFA for 10 min at room temperature. Cell monolayers and paraffin sections were then incubated with 0.1% Triton X-100 (Carl Roth) in PBS for 10 min at room temperature to allow permeabilization before blocking the samples with 3% Bovine Serum Albumin and 1% Normal Goat Serum (both Sigma-Aldrich) in PBS (BSA/NGS) for 1 h at room temperature. Antigens were detected by incubating with primary antibodies as shown in Supplementary Table S1 overnight at 4°C, followed by application of species specific Alexa Fluor 488 (Invitrogen, Thermo Fisher Scientific, Waltham, MA, United States) or Cy3 (Dianova, Hamburg, Germany) conjugated secondary antibodies. Concerning the cell monolayers, the chamber was removed and the coverslip overcast with Vectashield HardSet Antifade Mounting Medium with DAPI (Biozol, Eching, Germany). After costaining with DAPI (Calbiochem, San Diego, CA, United States), paraffin sections were dehydrated in ascending ethanol solutions and Xylene before covering with Entellan new (Merck Millipore). Representative immunofluorescent images were acquired using the Microscope Axio Imager. M2 with ApoTome.2 (Carl Zeiss, Oberkochen, Germany) and Fiji (ImageJ, National Institutes of Health, Bethesda, MD, United States) (Meir et al., 2015; Meir et al., 2019; Burkard et al., 2021; Meir et al., 2021; Nagler et al., 2022).

#### 2.3.4 Cell viability assays

To determine cell viability, CellTiter-Glo 2.0 Cell Viability Assay was used for Caco2 monolayers as well as 2D intestinal organoids and CellTiter-Glo 3D Cell Viability Assay was used for 3D intestinal organoids (both Promega, Madison, WI, United States). Samples were cultured in 96 multiwell plates and incubated with each Cell Viability Assay reagent respectively for 10 min under constant shaking and light protection. After resting for 25 min at room temperature, ATP-dependent luminescence was measured by GENios Pro (Tecan, Maennedorf, Switzerland) and results were normalized to untreated controls (Meir et al., 2019; Meir et al., 2021; Nagler et al., 2022).

#### 2.3.5 Dispase-based enterocyte dissociation assay

2D and 3D intestinal organoids were cultured in multiwell plates and incubated with cytomix as described above. For 3D culture, images were taken to assess the number of organoids per well at baseline. For 2D intestinal organoids, organoid culture was continued until confluency was achieved. Samples were washed with PBS and incubated with 2.4 U/mL Dispase-II (Roche, Basel, Switzerland) in HBSS for 30 min at 37°C to release the Caco2 monolayer from the well bottom and to dissolve the gel around the organoids. Afterwards, the samples were exposed to shear stress by pipetting up and down 5 times. Images of the whole wells were taken with fluorescence microscope BZ-9000 (Keyence, Osaka, Japan) and numbers of cell fragments before and after the experiments were quantified with Fiji (Schlegel et al., 2010; Spindler et al., 2015; Meir et al., 2019; Burkard et al., 2021; Nagler et al., 2022).

#### 2.3.6 Transepithelial electrical resistance

To evaluate TER, cell monolayers on electrode arrays or ThinCert cell culture inserts were measured using Electric Cell Substrate Impedance Sensing Z-Theta (Applied Biophysics) and Midi40 CO_2_ incubator (Thermo Scientific). 2D cell monolayers were cultured as described above and measurements started when reaching confluency after serum deprivation of Caco2 cells or differentiation of intestinal organoids respectively. Changes of TER where normalized to initial results (Schlegel et al., 2010; Meir et al., 2015; Meir et al., 2019; Burkard et al., 2021; Meir et al., 2021; Nagler et al., 2022).

#### 2.3.7 Statistical analysis

Data are presented as means ± Standard Error of the Mean (SEM). Statistical analysis was performed using Prism (GraphPad, La Jolla, CA, United States) and statistical significance was assumed for *p* < 0.05. Statistical significance is indicated in Figures by asterisks, * for *p* < 0.05, ** for *p* < 0.01, *** for *p* < 0.001 and **** for *p* < 0.0001. To determine normal distribution, either Shapiro-Wilk or D’Agostino and Pearson test was used depending on sample size. Accordingly, parametric data were analyzed using unpaired *t*-test or Ordinary one-way ANOVA while non parametric data were analyzed using Mann-Whitney or Kruskal–Wallis test respectively. The number of experiments carried out for each condition is indicated in the figure legends for respective experiments.

## 3 Results

### 3.1 Cytokine-induced loss of epithelial barrier function is associated with reduced transepithelial electrical resistance and changes of barrier proteins at the cell borders in Caco2 cells

**FIGURE 1:**
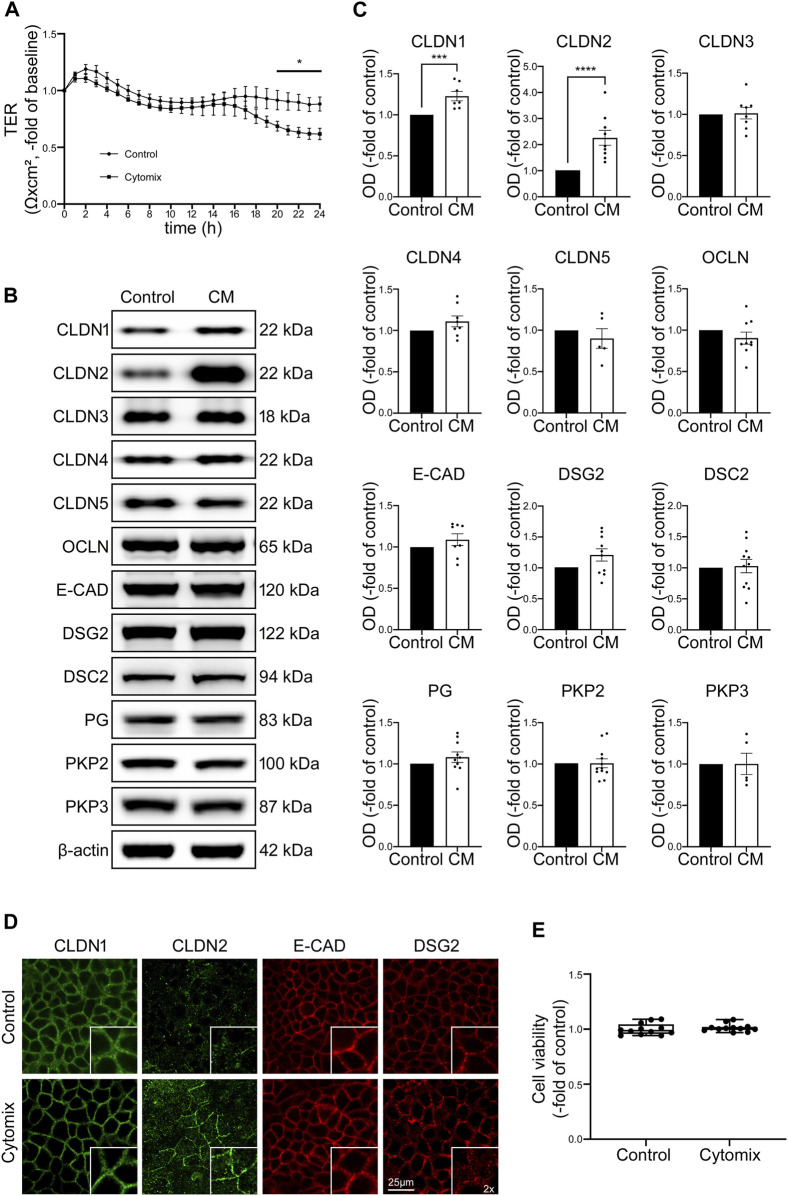
Changes in Caco2 cells after application of cytomix (CM) do not resemble typical features of patients with intestinal inflammation. **(A)** TER of Caco2 cells on electrode arrays shows significant reduction after 20 h of CM treatment, reaching a plateau after 24 h compared to untreated controls (*n* = 12). **(B)** Representative Western blots of CM treated Caco2 cells present stronger bands for CLDN1 and CLDN2 while all the other junctional proteins show now obvious changes. **(C)** Western blot quantifications present data as relative protein levels compared to β-actin (OD of protein of interest/OD of β-actin) normalized to untreated controls. Quantification confirms increased levels of CLDN1 and CLDN2 after CM incubation for 24 h. No significant changes of protein levels are detected for all other analysed junctional proteins. (n ≥ 5) **(D)** Immunofluorescence stainings of Caco2 cells show increased staining of CLDN2 at cell borders after CM application and no alterations of CLDN1, E-CAD and DSG2 (n = 6). **(E)** Cell viability assay presents no changes after CM treatment (n = 12). Data is presented as mean ± SEM and significant changes with asterisk (*p < 0.05, **p < 0.01, ***p < 0.001 and ****p < 0.0001).

To mimic inflammatory conditions in the models used for this study, cytomix containing TNFα, IFN-γ and IL-1β was applied to epithelial monolayers (Liu et al., 2006; Meir et al., 2015) in order to investigate the impact of inflammatory stimuli on functional and structural properties of the IEB. Application of cytomix to Caco2 monolayers led to reduced TER beginning after 20 h when TER was significantly decreased to 0.69 ± 0.04-fold of baseline (*p* = 0.0243) and finally reached a plateau at 0.62 ± 0.05-fold of baseline after 24 h (Figure 1A). Since the loss of several junctional proteins in patients suffering from intestinal inflammation was reported before (Spindler et al., 2015; Luissint et al., 2016; Meir et al., 2020), potential changes of junctional proteins were assessed using Western blot. In the Caco2 model none of the barrier-sealing junctional proteins, such as OCLN, E-cadherin (E-CAD) and desmosomal proteins Desmoglein2 (DSG2) and Desmocollin2 (DSC2) were changed following incubation of Caco2 cells with cytomix for 24 h. Only the tight junction protein Claudin1 (CLDN1) and pore-forming tight junction protein Claudin2 (CLDN2) were significantly increased compared to controls, while all other junctional proteins showed no relevant alterations in Western blot analysis (Figures 1B, C). When immunostaining of Caco2 monolayers was performed, DSG2 showed reduced staining patterns at the cell borders whereas CLDN2 was augmented following incubation with cytomix (Figure 1D). Analysis of cell viability of Caco2 cells after incubation with cytomix showed no significant changes compared to controls (Figure 1E). In summary, these data confirm that cytokines induce moderate loss of barrier function in Caco2 cells. While this supports the relevance of this traditional cell culture-based model, it is also obvious that these changes occur late after application of cytomix and typical loss of junctional protein levels and cell death seen in patients with intestinal inflammation was not observed (Meir et al., 2020).

### 3.2 Differentiated human intestinal organoids develop barrier properties comparable to the situation observed in healthy patients

**FIGURE 2:**
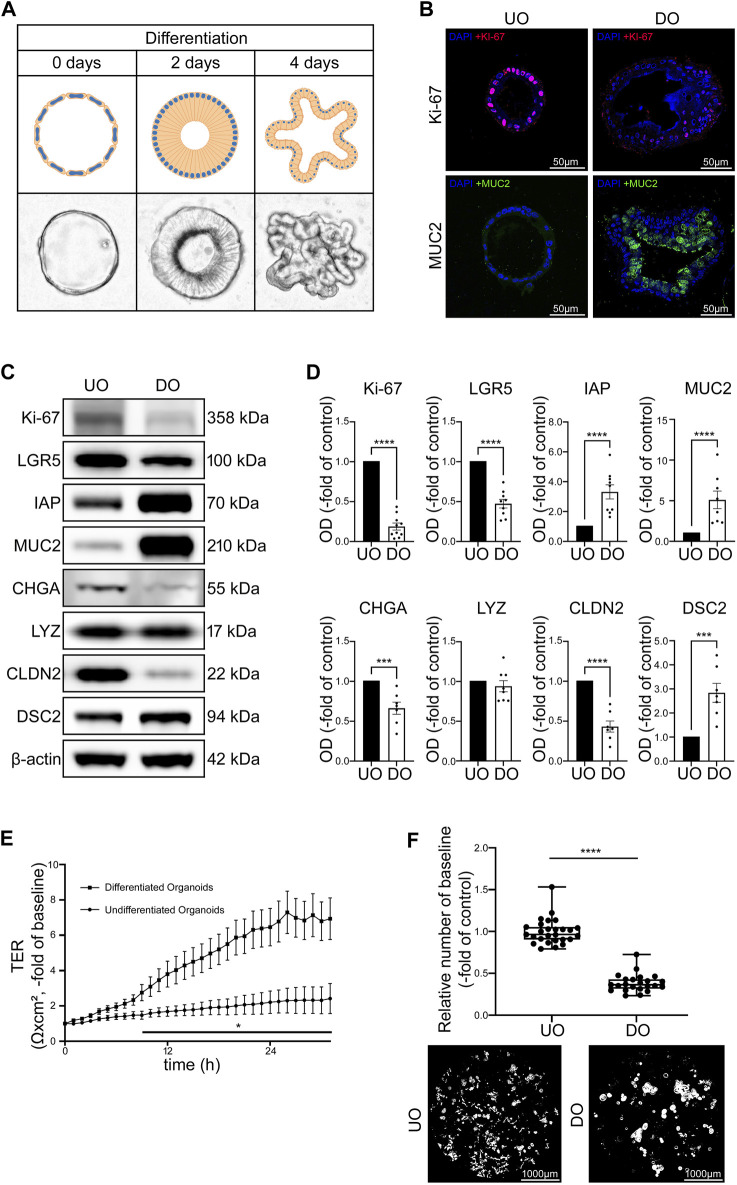
Differentiation of intestinal organoids shows maturation of IEB function and composition. **(A)** Schematic and microscopic display of progress of differentiation after 2 and 4 days. Undifferentiated intestinal organoids are thin walled and vesicular. During differentiation, the cells first transform into columnar cells and the organoid finally forms crypts and villi. **(B)** Immunofluorescence stainings of undifferentiated and 4 days differentiated organoids show decreased amount of Ki-67 positive cells and increased amount of MUC2 in differentiated organoids (n = 4 from 4 different patients). **(C)** Representative Western blots show weaker bands for Ki-67, LGR5, CHGA and CLDN2 in 4 days differentiated organoids while bands for IAP, MUC2 and DSC2 were stronger when compared to undifferentiated controls. **(D)** Western blot quantification shows decreased protein levels of Ki-67, LGR5, CHGA and CLDN2 while IAP, MUC2 and DSC2 presented increased levels after differentiation for 4 days. LYZ was not altered through differentiation. (n ≥ 7 from 6 different patients at different passages) **(E)** Differentiation of 2D intestinal organoids on electrode arrays resulted in significant increase of electrical resistance starting after 9 h compared to undifferentiated organoids and reached a plateau after 24 h (n = 12 from 4 different patients at different passages). **(F)** Dispase-based enterocyte dissociation assays show reduced fragmentation of organoids after 4 days of differentiation compared to undifferentiated controls (n = 23 from 4 different patients at different passages). Data is presented as mean ± SEM and significant changes with asterisk (*p < 0.05, **p < 0.01, ***p < 0.001 and ****p < 0.0001).

Next, we used undifferentiated (UO) and differentiated (DO) human intestinal organoids to characterize an improved model for intestinal barrier research by evaluating functional and structural features. We used human intestinal organoid cultures without differentiation media representing conditions of proliferation as typically seen in intestinal crypts. In addition, we tested different differentiation protocols to achieve a differentiated pattern of cells as can be found along the crypt-villus axis and in the villi. Following WNT-depletion, we found that differentiation patterns in terms of morphology, typical differentiation markers and intestinal barrier proteins was most comparable to conditions usually observed along the crypt-villus axis and the villi (Yin et al., 2014). Microscopy of organoids in culture following WNT-depletion revealed a transformation of thin walled, round, proliferative organoids into organoids that first develop a columnar epithelium and finally show crypt-villus formations during this differentiation process (Figure 2A). Immunostaining of specific cell markers verified increased differentiation as revealed by an overall reduction and redistribution of Ki-67-positive cells in the basolateral parts of the differentiated organoids suggesting the formation of crypt-villus-like structures. At the same time the overall number of goblet cells was increased during differentiation (Figure 2B). Similarly, Western blot analysis showed decreased levels of proliferation marker Ki-67 and reduced levels of intestinal stem cell marker leucine rich repeat containing G protein-coupled receptor 5 (LGR5) following WNT-depletion as well as an increase of enterocyte marker intestinal alkaline phosphatase (IAP) and goblet cell marker Mucin2 (MUC2). Chromogranin A (CHGA), a marker for enteroendocrine cells, was reduced following differentiation while Paneth cell marker lysozyme presented no changes. Analysis of barrier proteins showed reduced levels of CLDN2 and increased levels of DSC2 after differentiation, suggesting a possible sealing of the IEB during differentiation (Figures 2C, D).

TER measurements of 2D organoid cultures without differentiation medium showed only a slight increase of TER over 30 h of measurements whereas organoids cultivated with the differentiation protocol showed a gradual increase of TER values within 24 h to 6.47 ± 1.07-fold of baseline levels before reaching a plateau which was maintained in the following time course (Figure 2E). In line with this, direct comparison of undifferentiated and differentiated organoids using Dispase-based enterocyte dissociation assays revealed that cell adhesion was significantly stronger after differentiation when organoid fragmentation in differentiated organoids was decreased to 0.39 ± 0.02-fold of undifferentiated controls (Figure 2F).

### 3.3 Cytokine-stimulated organoid cultures display differential changes without differentiation

**FIGURE 3:**
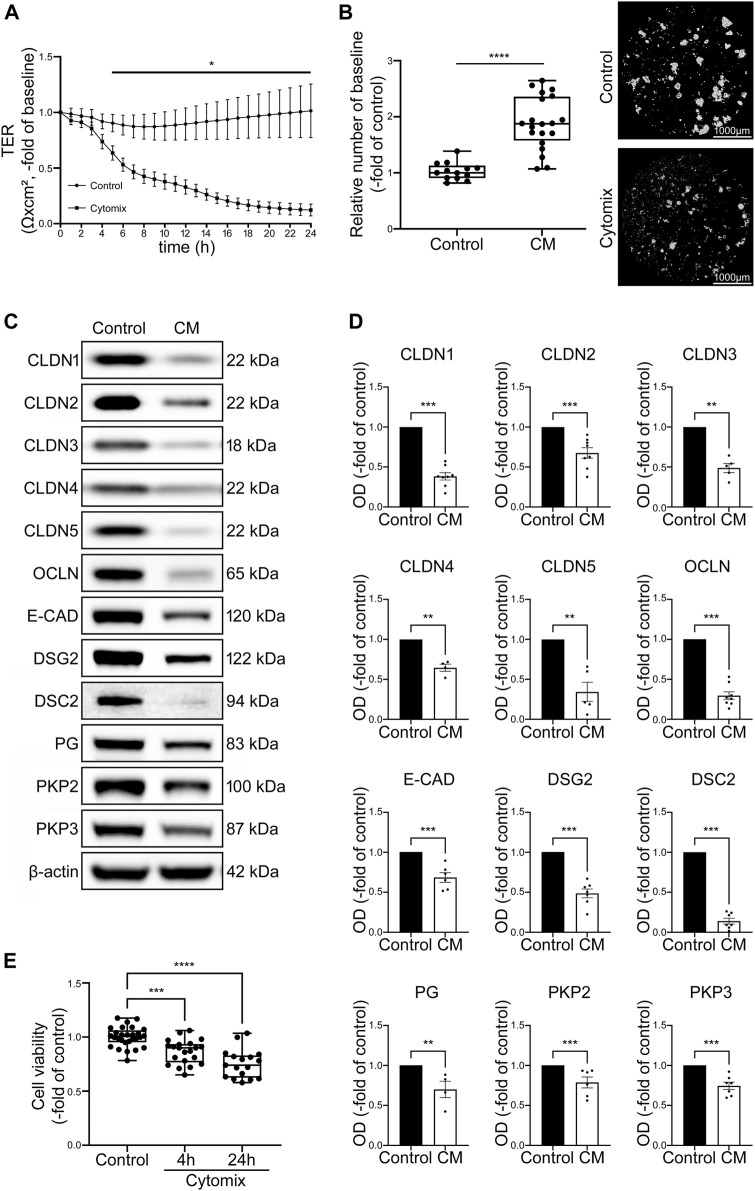
CM treatment of undifferentiated organoids leads to overall decrease of junctional proteins and reduced barrier function. **(A)** TER of 2D intestinal organoids on cell culture inserts shows significant reduction in CM treated organoids beginning after 5 h and dropped to 0.20 ± 0.07-fold of baseline after 24 h of CM incubation compared to controls (n = 12 from 5 different patients at different passages). **(B)** Dispase-based enterocyte dissociation assays show increased fragmentation of CM treated undifferentiated organoids compared to untreated controls (n = 20 from 4 different patients at different passages). **(C)** Representative Western blots show decreased levels of all junctional proteins after CM challenge. **(D)** Western blot quantification presents significant reduction of protein levels of all junctional proteins after CM treatment for 24 h (n ≥ 4 from 6 different patients). **(E)** Cell viability assay shows significant reduction of cell viability after 4h and 24 h of CM incubation (n = 17 from 4 different patients). Data is presented as mean ± SEM and significant changes with asterisk (*p < 0.05, **p < 0.01, ***p < 0.001 and ****p < 0.0001).

To investigate possible IEB changes after inflammatory stimuli, proliferative organoid cultures without differentiation media representing crypt-like conditions were incubated with cytomix. TER measurements carried out in 2D organoids showed a significant loss of TER values to 0.20 ± 0.07-fold of baseline after 24 h of incubation with cytomix (Figure 3A). In Dispase-based enterocyte dissociation assays the number of fragments was significantly increased to 1.89 ± 0.11-fold of controls after 24 h of incubation with cytomix (Figure 3B).

Western blot analysis revealed a significant reduction of tight junction proteins CLDN1, CLDN2, Claudin3 (CLDN3), Claudin4 (CLDN4), Claudin5 (CLDN5) and OCLN. Similarly, levels of adherens junction protein E-CAD and desmosomal proteins DSG2, DSC2 as well as desmosomal plaque proteins Plakoglobin (PG), Plakophilin2 (PKP2) and Plakophilin3 (PKP3) were decreased (Figures 3C, D). To exclude that this was predominately induced by cell death in response to cytomix application, cell viability assays were carried out after 4 h when the first drop of TER values became evident and after 24 h when barrier functions appeared completely lost. Cell viability was 86.48% ± 0.02% and 75.39% ± 0.04% of controls after 4 and 24 h of cytomix treatment respectively, demonstrating that particularly at the beginning of the barrier changes, no relevant amount of cell death was evident (Figure 3E).

With the reduction of virtually all junctional proteins though cell viability was only slightly reduced, the typical pattern of a differential loss of tightening tight junction proteins and increase of pore-forming tight junction proteins usually observed in patients with intestinal inflammation was not present. Together with the characterization patterns described above, we concluded that intestinal organoids as used under these conditions do not show the typical features of the inflammatory phenotype seen in patients with intestinal inflammation. Therefore, we tested whether the use of differentiated intestinal organoids represents a superior model.

### 3.4 Differentiated organoids represent an optimized *ex-vivo* model to mimic intestinal barrier changes in inflammation

**FIGURE 4:**
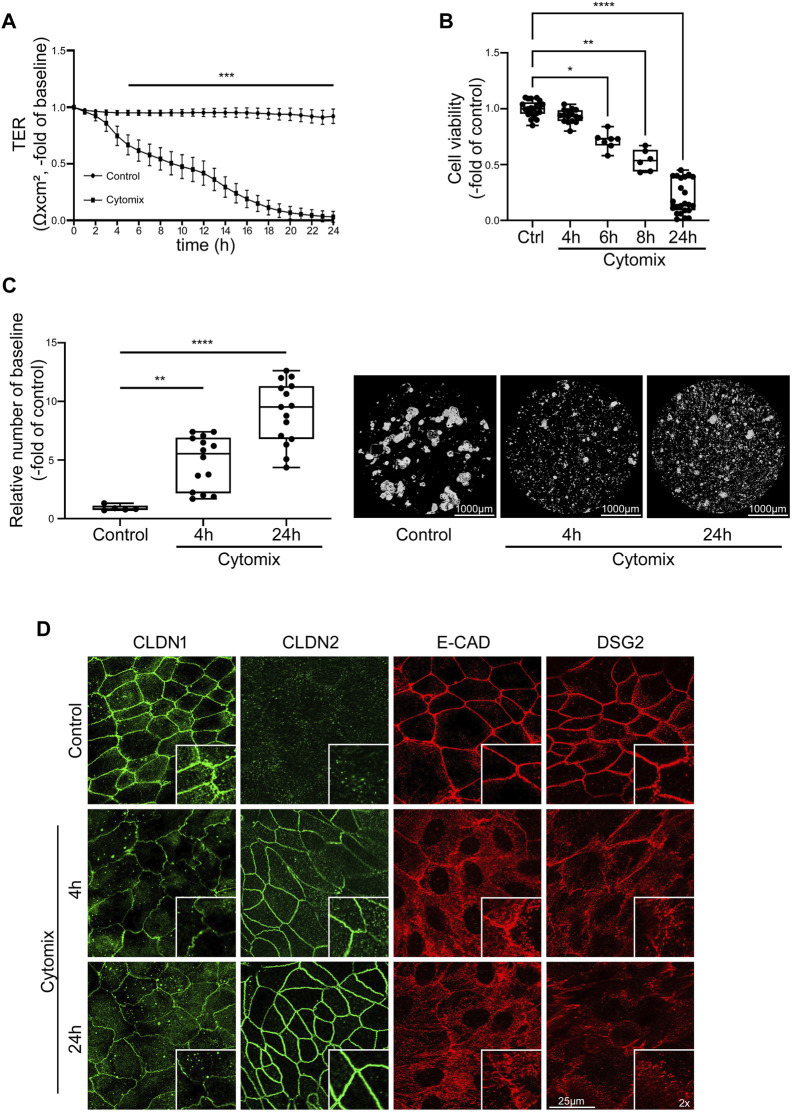
Inflammatory stimuli induce IEB dysfunction and disruption in differentiated intestinal organoids. Differentiation was executed for 4 days for all conditions shown here. **(A)** TER of 2D intestinal organoids on cell culture inserts shows significant reduction after 5 h of CM application compared to controls which further decreased until exhaustive barrier breakdown after 24 h of CM treatment (n = 17 from 6 different patients at different passages). **(B)** Cell viability reveals gradual decrease after CM treatment with significant reduction starting after 6 h (n ≥ 6 from 5 different patients at different passages). **(C)** Dispase-based enterocyte dissociation assay shows increased fragmentation after 4 and 24 h of CM incubation (n = 14 from 4 different patients at different passages). **(D)** Immunofluorescence staining of 2D intestinal organoids show reduced levels of CLDN1 after 4 and 24 h of CM incubation, increased staining for CLDN2 after 4 and 24 h of CM treatment and intracellular dislocation and reduction of E-CAD and DSG2 after 4 and 24 h of CM application (n = 3 from 3 different patients). Data is presented as mean ± SEM and significant changes with asterisk (*p < 0.05, **p < 0.01, ***p < 0.001 and ****p < 0.0001).

In order to mimic the impact of inflammation on mature intestinal epithelium, differentiated intestinal organoids were incubated with cytokines. Application of cytomix to differentiated 2D organoids resulted in profound loss of TER beginning after 4 h in differentiated organoids. In differentiated organoids, TER was significantly reduced to 0.67 ± 0.09-fold of baseline after 5 h and continuously dropped before levelling at <0.1 ± 0.05-fold of baseline after 24 h of incubation with cytomix (Figure 4A). To test for a critical contribution of cell death to the strong effect of cytomix on epithelial barrier function, we assessed cell viability at different time points after cytokine application, i.e., when loss of TER started to drop considerably after 4 h. Cell viability assay showed no alterations in the first hours of cytomix application (93.44% ± 1.48% after 4 h) compared to controls. However, at later time points, a gradual decrease of viable cells was evident beginning after 6 h which finally settled at 20.26% ± 3.14% of controls after 24 h. This suggested that differentiated organoids have increased susceptibility to cytomix when compared to undifferentiated organoids (Figure 4B). Interestingly, reduced concentrations of cytokines showed comparable effects on cell viability (not shown). This suggested that loss of barrier function cannot be explained by the induction of cell death alone at earlier time points. Therefore, we tested additional features contributing to loss of barrier function that had been reported before in detail. First, we assessed the extent of reduced intercellular adhesion following cytomix application after 4 and 24 h using Dispase-based enterocyte dissociation assays. After 4 h, cell adhesion was significantly reduced as revealed by an increased amount of fragmentation of 5.82 ± 0.80-fold of controls which was further augmented after 24 h to 10.65 ± 1.0-fold of controls (Figure 4C). Immunostaining of junctional proteins was carried out after 4 and 24 h to document the changes in enterocyte monolayers. We observed a reduction of CLDN1 after 4 and 24 h of cytomix treatment, while CLDN2 showed a gradually increasing staining pattern at the two time points. Immunostaining of E-CAD and DSG2 showed an overall reduction of the staining pattern as well as a redistribution from the cell borders into the cytoplasm (Figure 4D).

**FIGURE 5:**
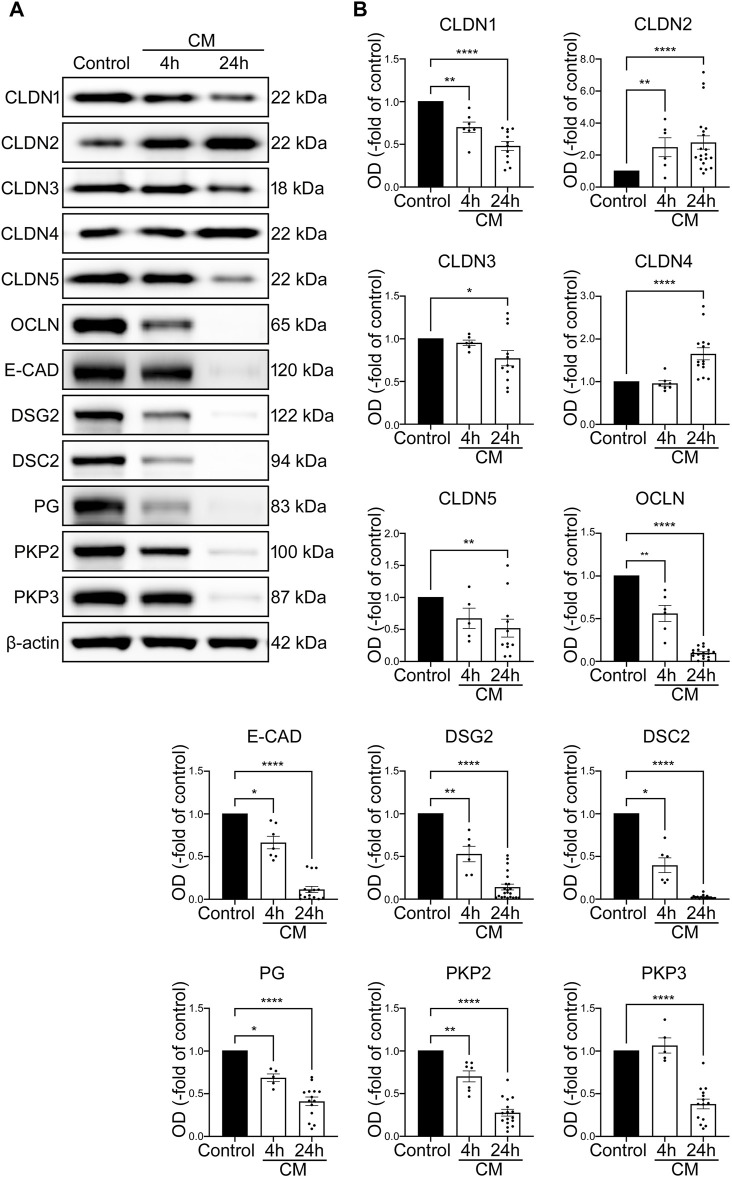
IEB changes of differentiated intestinal organoids under inflammatory conditions resemble the IBD phenotype. Differentiation was executed for 4 days for all conditions shown here. **(A)** Representative Western blots show gradually diminished bands of CLDN1, CLDN5, OCLN, E-CAD, DSG2, DSC2, PG and PKP2 after 4 and 24 h of CM application, smaller bands of CLDN3 and PKP3 after 24 h of CM incubation and increase of CLDN2 after 4 and 24 h and CLDN4 after 24 h of CM treatment. **(B)** Western blot quantification reveals gradually decreased protein levels after 4 and 24 h of CM incubation for CLDN1, CLDN5, OCLN, E-CAD, DSG2, DSC2, PG and PKP2. Reduction of protein levels after 24 h was observed for CLDN3 and PKP3. Increase of CLDN2 was revealed after 4 and 24 h of CM treatment and augmented protein levels of CLDN4 after 24 h of CM application. (n = 5 from 5 different patients). Data is presented as mean ± SEM and significant changes with asterisk (*p < 0.05, **p < 0.01, ***p < 0.001 and ****p < 0.0001).

In addition, loss of junctional proteins was assessed after 4 h and after 24 h using Western blotting. At both time points, Western blot analysis revealed decreased protein levels of barrier sealing tight junction proteins CLDN1, CLDN3, CLDN5, OCLN, reduction of adherens junction protein E-CAD as well as desmosomal proteins DSG2 and DSC2 and the desmosomal plaque proteins PG, PKP2 and PKP3. According to the reduced cell viability, the changes were in general more pronounced after 24 h compared to the situation after 4 h following application of cytomix. Pore forming tight junction protein CLDN2 was increased after 4 and 24 h of cytomix incubation. In addition, the barrier sealing tight junction protein CLDN4 was augmented (Figures 5A, B).

### 3.5 Functional and quantitative assessments of 2D and 3D intestinal organoids are comparable

**FIGURE 6:**
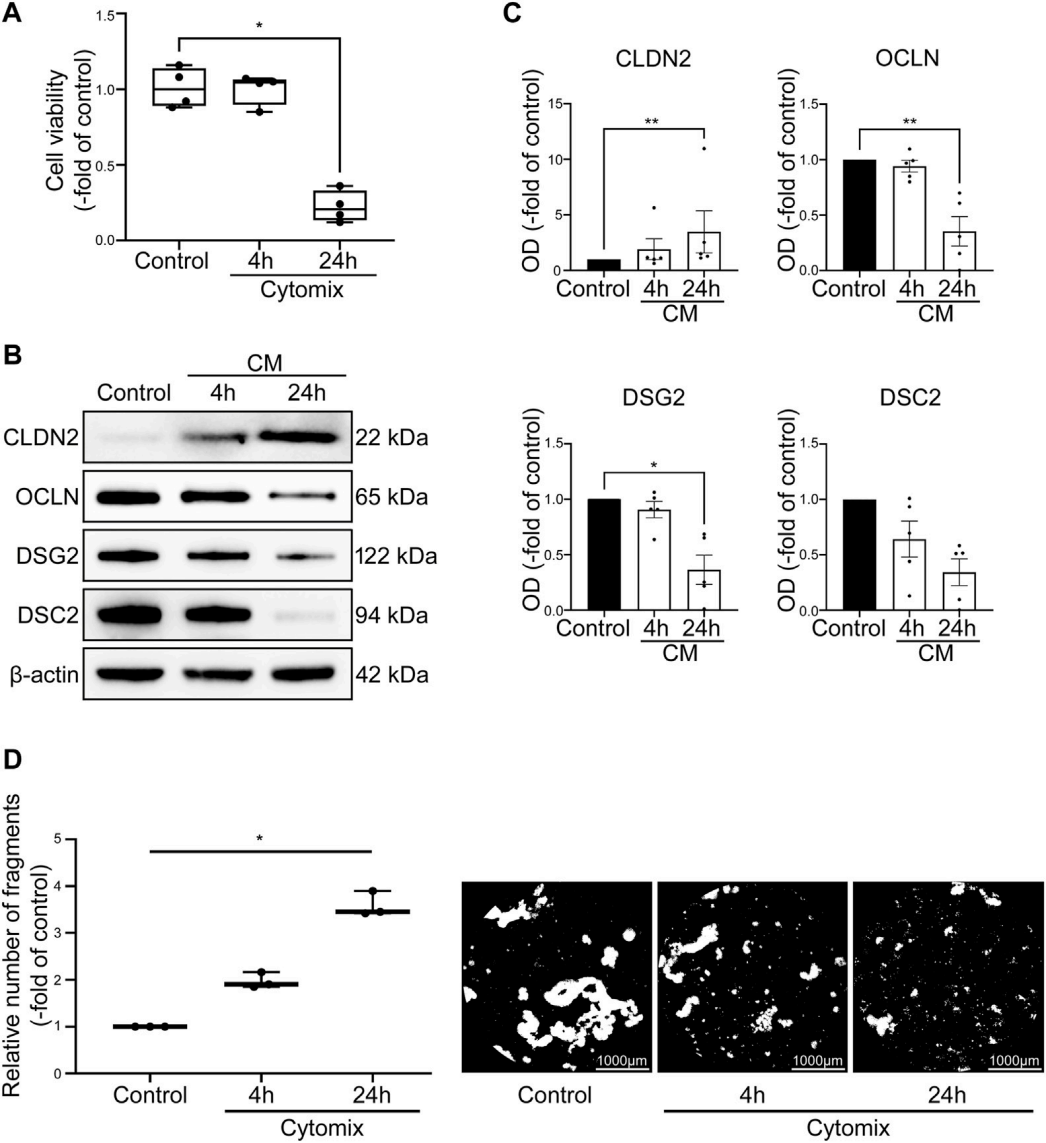
Cytomix application causes comparable effects on 2D intestinal organoids compared to 3D intestinal organoids. Differentiation was executed for 4 days for all conditions shown here. **(A)** Cell viability is not changed after 4 h of CM application but shows significant reduction of viable cells after 24 h of CM treatment (n = 4 from 4 different patients). **(B)** Representative Western blots show increased levels of CLDN2 after CM incubation and reduction of OCLN, DSG2 and DSC2. **(C)** Western blot analyses reveal augmented levels of CLDN2 after 4 and 24 h of CM application while barrier sealing proteins DSG2, DSC2 and OCLN are gradually decreased (n ≥ 5 from 6 different patients). **(D)** CM incubation leads to gradually increased fragmentation after 4 and 24 h as revealed by Dispase-based enterocyte dissociation assays (n = 3 from 3 different patients). Data is presented as mean ± SEM and significant changes with asterisk (*p < 0.05, **p < 0.01, ***p < 0.001 and ****p < 0.0001).

To ensure that intestinal 2D and 3D cultured organoids are comparable and thus will react equally to inflammatory stimuli, we executed comparative investigations of 3D methods in 2D organoids. Application of cytomix on 2D intestinal organoids did not result in changes of cell viability after 4 h (100.30% ± 5.12%) but showed significant decline of viable cells after 24 h (22.25% ± 5.20%) (Figure 6A). Dispase-based enterocyte dissociation assays of 2D intestinal organoids revealed gradual loss of cell adhesion in the time course of cytomix incubation with increased fragmentation to 1.97 ± 0.10-fold of control after 4 h and further augmentation to 3.59 ± 0.15-fold of control after 24 h of inflammatory stimuli (Figure 6D). Quantitative analysis of selected barrier proteins showed decreased levels of barrier sealing proteins DSG2, DSC2 and OCLN while CLDN2 was increased after cytomix application for 4 and 24 h (Figures 6B, C). In summary, the comparative investigations of 2D intestinal organoids showed congruent responses to cytokine stimulation than 3D intestinal organoids.

## 4 Discussion

The present study extends previous studies in further refining and validating a human *ex-vivo* model of the intestinal epithelial barrier with a profound characterization of cytokine-induced changes on junctional proteins in different models. Usually, the human intestinal epithelial barrier is not accessible for cellular analysis, which makes human intestinal organoid cultures most interesting to enable a further mechanistic analysis. This will allow comparisons and correlations with the well-characterized features of the intestinal barrier in the *in-vivo* situation both under basal conditions and in inflammation. Although it has been widely assumed that intestinal organoid cultures may be useful tools, detailed protocols to specifically assess the aspect of barrier function and cytokine-induced changes in detail were lacking (Yoo and Donowitz, 2019; Xu et al., 2021; Lechuga et al., 2023). This is now substantiated by our current comparison of a traditionally used cancer-derived intestinal cell line (Caco2 cells), which has been used for intestinal barrier research and human intestinal organoid cultures at different stages of differentiation. Here, we recapitulate that Caco2 cells display only some of the typical features seen in intestinal inflammation with the advantages of easy availability. However, our data from intestinal organoids represent the typical features previously described to occur in intestinal inflammation including loss of intercellular adhesion, redistribution and loss of junctional proteins resulting in loss of barrier function due to reduced cell junctions at the cell borders finally leading to cell death. These changes are represented most comprehensively in differentiated organoids. In summary, this represents a long-awaited prerequisite to further assess the molecular mechanisms of inflammation-induced loss of intestinal epithelial barrier function.

### 4.1 Cancer-derived cells lines show limitations for intestinal barrier research

Hidalgo et al. published a characterization of the barrier competence of the Caco2 cell line in 1989, showing that these cells form a polarized monolayer that exhibits barrier functions such as selective permeability and transepithelial electrical resistance (Hidalgo et al., 1989). Caco2 cells also present enterocyte-like differentiation and polarization with the ability to express junctional complexes (Pageot et al., 2000). Therefore, Caco2 cells have been widely used for quantitative and functional intestinal research (Suzuki et al., 2009; Schlegel et al., 2010; Schlegel et al., 2011; Zheng et al., 2011; Kaak et al., 2022). Still, the transferability of the results especially on findings of cellular signalling were continuously challenged due to the cancerous origin of the cell line. This is further substantiated by the current observation that inflammatory challenge did not result in any changes of cell viability which is commonly observed in the *in-vivo* situation (Koch and Nusrat, 2012) and in the organoid model presented here. This confirms that Caco2 cells possess non-physiological resilience to inflammatory stimuli. Accordingly, cytokine application only resulted in minor changes of TER, which is supported by previous observations of a stronger cell-cell tightness of Caco2 cells than small intestine *in-vivo* (Lopez-Escalera and Wellejus, 2022). Most importantly, the cell line does not exhibit classic changes of junctional proteins under inflammatory conditions. Again, this is not comparable to tissue specimens from IBD patients, where substantial changes of junctional proteins depending on the extent of inflammation were reported (Gassler et al., 2001; Kucharzik et al., 2001; Luissint et al., 2016; Meir et al., 2020). On the other hand, these cells keep undisputable advantages such as easy availability, low costs, easy handling and high reproducibility of experimental set-ups. Following this, the use of the Caco2 cell line is still warranted when keeping the extensive limitations in mind.

### 4.2 Differentiated organoids share typical features of an intact intestinal barrier

Intestinal organoids in expansive culture resemble the stem cell niche *in-vivo*. They present high rates of proliferation and a relatively low intercellular adhesion possibly due to the need for constant mitosis. This is supported by our present data in which intercellular adhesion as revealed by Dispase-based enterocyte dissociation assays was augmented in differentiated intestinal organoids. Controlled differentiation of intestinal organoids by WNT-depletion leads to a morphological transformation from crypt-like, highly proliferative organoids into organoids with differentiated epithelium with crypt-villus formations. In line with previously described differentiation protocols, WNT-depletion resulted in maturation of the intestinal organoids into different cell types resembling intestinal mucosa (Yin et al., 2014). The successful differentiation in our current model is confirmed by the loss of intestinal epithelial stem cell marker LGR5 and reduced proliferation rates shown by a reduction of Ki-67. Furthermore, IAP representing an enterocyte marker (Hinnebusch et al., 2004; Yin et al., 2014) and MUC2 representing goblet cells (Yin et al., 2014; Liu et al., 2023) were increased whereas CHGA representing enteroendocrine cells (Yin et al., 2014; Massironi et al., 2016) and CLDN2 were reduced in the time course of differentiation. In the *in-vivo* situation, CLDN2 is predominately found in the crypts and is usually upregulated in intestinal inflammation (Lu et al., 2013; Luettig et al., 2015; Landy et al., 2016; Luissint et al., 2016; Meir et al., 2020). Finally, these changes correlated with an augmented barrier function as revealed by increased TER values and augmented protein expression of tightening junctional proteins following WNT-depletion which further underlines the conclusion that differentiation of the organoid model represented here was successful. In summary, it may be assumed that differentiated human intestinal organoids develop barrier properties comparable to the situation observed in healthy patients whereas immature organoids maintain many features usually found within the crypts. To our knowledge, the organoid model has not been characterized to that extent with respect to barrier function which is the prerequisite to further assess the effect of pro-inflammatory cytokines.

### 4.3 Immature organoids appear not ideal for intestinal barrier assessment under basal conditions and in response to inflammatory stimuli

Intestinal crypts are characterized by highly proliferating stem cells embedded in the stem cell niche with the ability for frequent cell division and migration along the crypt-villus axis. This condition appears to be represented by undifferentiated or immature intestinal organoid cultures. Organoids under these conditions show low intercellular adhesion and do not exert good barrier properties. The reduced cell-cell adhesion is documented by the fact that enterocyte dissociation assays show an increased rate of fragmentation and no functional maturation of the immature intestinal epithelial barrier as demonstrated by persistently low TER values, although relevant proteins to mediate cell-cell adhesion such as E-CAD and desmosomal proteins DSG2 and DSC2 as well as tight junctions for barrier sealing are clearly present. This suggests that despite the presence of junctional proteins, barrier maturation does not occur under proliferative culture conditions. This is important because these conditions do not allow conclusions on barrier functions and underlying signalling pathways. Rather, these conditions in comparison with differentiated organoids will help to further refine the detailed mechanisms of barrier differentiation along the crypt-villus axis. This is further supported by the experiments using cytomix to mimic the conditions of intestinal inflammation. In general, specific analyses of the situation in the crypts are rare but it has been suggested that cytokines predominately induce cell death in this compartment (Patankar and Becker, 2020). This appeared not to be case in our experiments, since cell viability was only slightly reduced by 20%–25% even after prolonged incubation of the organoids with cytokines. Rather, after cytokine challenge, immature intestinal organoids showed decreased levels of all barrier proteins investigated resulting in further impaired cell-cell adhesion and consecutive loss of TER. The overall reduction of all junctional proteins tested does not correspond to the changes of junctional proteins usually observed in intestinal inflammation (Luettig et al., 2015; Landy et al., 2016; Luissint et al., 2016; Meir et al., 2020). In view of the overall limited barrier properties and intercellular adhesion, we assume that it is important to use differentiated organoids to get a more reliable model of the *in-vivo* situation for further analysis of molecular mechanisms of barrier dysfunction in inflammation.

### 4.4 Differentiated intestinal organoids present a suitable model for the investigation of inflammation-induced intestinal epithelial barrier dysfunction

The use of differentiated intestinal organoids revealed that they were more susceptible in response to pro-inflammatory cytokines. Loss of cell viability and reduced intercellular adhesion were more pronounced when compared to immature organoids. The changes of barrier function and barrier proteins in the differentiated organoid model following cytokine application observed here, closely resemble the situation observed in patients suffering from intestinal inflammation: We observed an overall reduction of barrier-sealing tight junction proteins CLDN1, CLDN3, CLDN5 and OCLN. Whereas pore-forming CLDN2, which is constantly seen to be upregulated in patients with intestinal inflammation, is increased (Luettig et al., 2015; Luissint et al., 2016; Barmeyer et al., 2017; Meir et al., 2020). In addition, we found that comparable to the observation made in patients so far that there is an early reduction of desmosomal proteins DSG2, DSC2 as well as of desmosomal plaque proteins PG and PKP2 following application of cytokines (Spindler et al., 2015; Meir et al., 2019; Meir et al., 2020). In summary, we assume from all these correlations that the use of differentiated organoids subjected to cytokines provides a reproducible and reliable human *ex-vivo* model for in-depth mechanistic analyses in the future. A promising alternative to the application of cytokines will be to establish co-cultures with immune cells to further assess the immune-epithelial interaction and in intestinal barrier regulation.

Besides the characterization of the model as shown here, an important and interesting observation is that loss of intercellular adhesion and redistribution of junctional proteins precedes loss of TER which is then accompanied by a gradual reduction of junctional proteins finally leading to reduced cell viability. This suggests that there is a hierarchical sequence finally leading to total loss of barrier function and cell death. This supports the notion that proper intercellular adhesion is required to maintain tight junction stability which are in the prerequisite for intact barrier properties. The early loss of intercellular adhesion and predominantly desmosomal proteins following application of cytokines may point to a pace-maker role of the intercellular adhesion proteins DSG2 and DSC2 to induce loss of barrier function in intestinal inflammation. A comparable role can be assumed for E-CAD which was also reduced in response to cytokine application. The close interaction between adherens junctions and desmosomes as well as a contribution of desmosomes to regulate tight junction integrity was recognized in previous studies (Rübsam et al., 2018; Burkard et al., 2021; Nagler et al., 2022). Furthermore, there is evidence for an interaction between DSC2 and cell-matrix adhesion, which was not tested here (Flemming et al., 2020). A potential mechanism underlying the induction of cell death as consequence of altered barrier function could be that cytokine-induced loss and fragmentation of DSG2 was shown to induce intestinal epithelial cell death by modulating Caspase3 activity (Nava et al., 2007; Cirillo et al., 2008). A comparable observation has been made for tight junction disruption and cell death in hepatocytes (Sonoi and Hagihara, 2021).

Profound cellular analysis will be required to further resolve the detailed cellular processes in the organoid model under inflammation. In summary, the present study provides a reproducible, well-characterized *ex-vivo* model which is required to further refine intestinal barrier research in human cells.

### 4.5 Both 2D and 3D differentiated intestinal organoids are suitable for intestinal barrier research

The analysis of 2D intestinal organoids in methods, that were primarily conducted in 3D intestinal organoids revealed congruent results to cytomix application. While the 2D intestinal organoids presented no changes in cell viability after 4 h of cytokine treatment, there was already loss of cell adhesion and initial changes in the composition of IEB proteins evident. After 24 h of cytomix incubation, there was significant cell death which was accompanied by more profound changes of cell adhesion in barrier composition. In summary, these results conform to the observations that were made in 3D organoid cultures, proving that the two culture forms of intestinal organoids react uniformly to inflammatory stimuli and can therefore both be used for functional and quantitative assessments.

## Data Availability

The original contributions presented in the study are included in the article/Supplementary Material, further inquiries can be directed to the corresponding author.
