# Supplementary material for: Human organoids are superior to cell culture models for intestinal barrier research
Source: Front Cell Dev Biol. 2023 Oct 2;11:1223032. doi: 10.3389/fcell.2023.1223032 (PMC10577213; doi:10.3389/fcell.2023.1223032)
Supplement: Supplementary file 1 [file DataSheet1.docx]

Supplementary Material

Human organoids are superior to cell culture models for intestinal barrier research

**Catherine Kollmann^1^, Hannah Buerkert^1^, Michael Meir^1^, Konstantin Richter^1^, Kai Kretzschmar^2^, Sven Flemming^1^, Matthias Kelm^1^, Christoph-Thomas Germer^1^, Christoph Otto^1^, Natalie Burkard^1^, Nicolas Schlegel^1^***

*** Correspondence:** Prof. Dr. Nicolas Schlegel: Schlegel_N@ukw.de

# Supplementary Table

Supplementary Table 1. List of antibodies used in this study with applying method and manufacturer with catalog number.

| Antibodies | Western blot | Immunofluorescence  staining |
| --- | --- | --- |
| Chromogranin A | Santa-Cruz, 393941 | - |
| Claudin1 | Invitrogen, 37-4900 | Invitrogen, PA5-32350 |
| Claudin2 | Invitrogen, 32-5600 | Invitrogen, 32-5600 |
| Claudin3 | Invitrogen, PA5-16867 | - |
| Claudin4 | Invitrogen, 32-9400 | - |
| Claudin5 | Invitrogen, 34-1600 | - |
| Desmocollin2 | Abcam, 95967 | - |
| Desmoglein2 | Invitrogen, 32-6100 | Santa-Cruz, 80663 |
| E-cadherin | BD Biosciences, 610182 | BD Biosciences, 610182 |
| Ki-67 | Abcam, 15580 | Abcam, 15580 |
| Lysozome | Abcam, 108508 | - |
| Intestinal alkaline phosphatase | Invitrogen, PA5-19987 | - |
| LGR5 | Invitrogen, UM800102 | - |
| Mucin2 | Santa-Cruz, 515032 | Santa-Cruz, 515032 |
| Occludin | Invitrogen, 33-1500 | - |
| Plakoglobin | Progen, 61005 | - |
| Plakophilin2 | Progen, 651101 | - |
| Plakophilin3 | Invitrogen, 35-7600 | - |

Supplementary Table 2. List of abbreviations used in the manuscript.

| Abbreviation | Meaning |
| --- | --- |
| 2D | Two-dimensional |
| 3D | Three-dimensional |
| BM | Basal medium |
| BSA | Bovine Serum Albumin |
| CLDN1 | Claudin1 |
| CLDN2 | Claudin2 |
| CLDN3 | Claudin3 |
| CLDN4 | Claudin4 |
| CLDN5 | Claudin5 |
| CM | Cytomix |
| DAPI | 4′,6-diamidino-2-phenylindole |
| DSC2 | Desmocollin2 |
| DSG2 | Desmoglein2 |
| DMEM | Dulbecco’s Modified Eagle’s Medium |
| DO | Differentiated organoids |
| E-CAD | E-cadherin |
| EDTA | Ethylenediaminetetraacetic acid |
| HBSS | Hank’s Balanced Salt Solution |
| HEPES | 4-(2-hydroxyethyl)-1-piperazineethanesulfonic acid |
| IBD | Inflammatory bowel disease |
| IEB | Intestinal epithelial barrier |
| IFNγ | Interferon-γ |
| IL-1β | Interleukin 1β |
| LGR5 | Leucine rich repeat containing G protein-coupled receptor 5 |
| NGS | Normal Goat Serum |
| OCLN | Occludin |
| OD | Optical density |
| PBS | Phosphate Buffered Saline |
| PFA | Formaldehyde solution |
| PG | Plakoglobin |
| PKP2 | Plakophilin2 |
| PKP3 | Plakophilin3 |
| SDS | Sodium dodecyl sulfate |
| SEM | Standard Error of the Mean |
| TER | Transepithelial electrical resistance |
| TNFa | Tumor necrosis Factor-α |
| UO | Undifferentiated organoids |

Supplementary Table 3. List of commercial products used in this study with manufacturer and catalog number.

| Commercial product | Manufacturer | Location of manufacturer | Catalog number |
| --- | --- | --- | --- |
| 12 well chambered coverslips | Ibidi | Graefelfing, Germany | 81201 |
| A83-01 | Tocris Bioscience, Bio-Techne | Bristol, UK | 2939 |
| Advanced DMEM/F-12 | Gibco, Thermo Fisher Scientific | Waltham, MA, USA | 12634-010 |
| Antibiotic-Antimycotic | Gibco, Thermo Fisher Scientific | Waltham, MA, USA | 15240-062 |
| B-27 (50x) w/o Vitamin A | Gibco, Thermo Fisher Scientific | Waltham, MA, USA | 12587-010 |
| Bovine Serum Albumin | Sigma-Aldrich | St. Louis, MO, USA | A4503 |
| Caco-2 cells | ATCC, LGC Standards | Wesel, Germany | HTB-37 |
| CellTiter-Glo 2.0 Cell Viability Assay | Promega | Madison, WI, USA | G9241 |
| CellTiter-Glo 3D Cell Viability Assay | Promega | Madison, WI, USA | G9681 |
| DAPI | Calbiochem | San Diego, CA, USA | 268298 |
| Dispase-II | Roche | Basel, Switzerland | 04942078001 |
| Dulbecco's Modified Eagle's Medium | Sigma-Aldrich | St. Louis, MO, USA | D5796 |
| Dulbecco’s Phosphate Buffered Saline | Gibco, Thermo Fisher Scientific | Waltham, MA, USA | 14190-094 |
| EDTA | Serva Electrophoresis | Heidelberg, Germany | 11278.02 |
| Electrode arrays | Applied Biophysics | Troy, NY, USA | 8W10E+ |
| Entellan new | Merck Millipore | Burlington, MA, USA | 1.07961.0500 |
| Ethanol | Carl Roth | Karlsruhe, Germany | K928.4 |
| Fetal Calf Serum | Gibco, Thermo Fisher Scientific | Waltham, MA, USA | 10270-106 |
| Formaldehyde solution 3,5% | Otto Fischar | Saarbruecken, Germany | 27244 |
| Geltrex LDEV-Free Reduced Growth Factor Basement Membrane Matrix | Gibco, Thermo Fisher Scientific | Waltham, MA, USA | A1413202 |
| Gentamicin | Genaxxon bioscience | Ulm, Germany | M3122.0050 |
| GlutaMAX | Gibco, Thermo Fisher Scientific | Waltham, MA, USA | 35050-061 |
| Glycerol | Sigma-Aldrich | St. Louis, MO, USA | G5516 |
| Growth Factor Reduced Basement Membrane Matrix | Corning | Corning, NY, USA | 356238 |
| Hank’s Balanced Salt Solution | Sigma-Aldrich | St. Louis, MO, USA | H9394 |
| HEPES | Gibco, Thermo Fisher Scientific | Waltham, MA, USA | 15630-056 |
| HistoGel | Thermo Scientific, Thermo Fisher Scientific | Waltham, MA, USA | HG-4000-012 |
| hR-Spondin-1 | PeproTech | Hamburg, Germany | 120-38 |
| Human epidermal growth factor | PeproTech | Hamburg, Germany | AF-100-15 |
| Hydrochloric acid | Carl Roth | Karlsruhe, Germany | 0281.1 |
| IFNγ | Merck Millipore | Burlington, MA, USA | IF002 |
| IL-1β | Sigma-Aldrich | St. Louis, MO, USA | IL038 |
| IntestiCult Organoid Growth Medium Human | Stemcell Technologies | Vancouver, Canada | 06010 |
| [Leu15]-Gastrin I human | Sigma-Aldrich | St. Louis, MO, USA | G9145-5MG |
| LY2157299 | Axon MedChem | Groningen, Netherlands | 1491 |
| mNoggin | PeproTech | Hamburg, Germany | 250-38 |
| N-2 (100x) | Gibco, Thermo Fisher Scientific | Waltham, MA, USA | 17502-048 |
| N-Acetyl-L-cysteine | Sigma-Aldrich | St. Louis, MO, USA | A9165-5G |
| Nicotinamide | Sigma-Aldrich | St. Louis, MO, USA | N0636 |
| Nitrocellullose membrane | Invitrogen, Thermo Fisher Scientific | Waltham, MA, USA | LC2009 |
| Nonfat dried milk powder | PanReac AppliChem | Darmstadt, Germany | T145.3 |
| Penicillin-Streptomycin | Gibco, Thermo Fisher Scientific | Waltham, MA, USA | 15140-122 |
| Pierce BCA Protein Assay Kit | Thermo Scientific, Thermo Fisher Scientific | Waltham, MA, USA | 23227 |
| Protease Inhibitor Cocktail 100x | Thermo Scientific, Thermo Fisher Scientific | Waltham, MA, USA | 78438 |
| SB202190 | Sigma-Aldrich | St. Louis, MO, USA | S7067-5MG |
| Sodium chloride | Sigma-Aldrich | St. Louis, MO, USA | 31434 |
| Sodium dodecyl sulfate | Carl Roth | Karlsruhe, Germany | CN30.2 |
|  |  |  |  |
| SuperSignal West Pico PLUS Chemiluminescent Substrate | Thermo Scientific, Thermo Fisher Scientific | Waltham, MA, USA | 34580 |
| ThinCert cell culture inserts | Greiner Bio-One | Kremsmuenster, Austria | 662640 |
| TNFα | Merck Millipore | Burlington, MA, USA | GF023 |
| Tris ultrapure | PanReac AppliChem | Darmstadt, Germany | A1086 |
| TrypLE Express Enzyme | Gibco, Thermo Fisher Scientific | Waltham, MA, USA | 12605-010 |
| Trypsin-EDTA | Sigma-Aldrich | St. Louis, MO, USA | T3924-100ML |
| Tween 20 | PanReac AppliChem | Darmstadt, Germany | A4974 |
| Vectashield HardSet Antifade Mounting Medium with DAPI | Biozol | Eching, Germany | H-1500 |
| Xylene AnalaR Normapur | VWR International | Radnor, PA, USA | 28975.360 |
| Y-27632 dihydrochloride | Tocris Bioscience, Bio-Techne | Bristol, UK | 1254 |
